# Weaning from Mechanical Ventilator in a Long-term Acute Care Hospital: A Retrospective Analysis

**DOI:** 10.2174/1874306402014010062

**Published:** 2020-12-18

**Authors:** Salim Surani, Munish Sharma, Kevin Middagh, Hector Bernal, Joseph Varon, Iqbal Ratnani, Humayun Anjum, Alamgir Khan

**Affiliations:** 1 Texas A&M University, Texas, USA; 2 Post Acute Medical Center, Corpus Christi, Texas, USA; 3 University of Houston, Houston, Texas, USA; 4 Houston Methodist Hospital, Houston, Texas, USA; 5 University of North Texas, Denton, Texas, USA

**Keywords:** Prolonged mechanical ventilation, Long term acute care, Ventilator weaning, Difficult weaning, Respiratory therapist in weaning, Liberation from mechanical ventilation

## Abstract

**Background:**

Prolonged Mechanical Ventilation (PMV) is associated with a higher cost of care and increased morbidity and mortality. Patients requiring PMV are referred mostly to Long-Term Acute Care (LTAC) facilities.

**Objective:**

To determine if protocol-driven weaning from mechanical ventilator by Respiratory Therapist (RT) would result in quicker weaning from mechanical ventilation, cost-effectiveness, and decreased mortality.

**Methods:**

A retrospective case-control study was conducted that utilized protocol-driven ventilator weaning by respiratory therapist (RT) as a part of the Respiratory Disease Certification Program (RDCP).

**Results:**

51 patients on mechanical ventilation before initiation of protocol-based ventilator weaning formed the control group. 111 patients on mechanical ventilation after implementation of the protocol formed the study group. Time to wean from the mechanical ventilation before the implementation of protocol-driven weaning by RT was 16.76 +/- 18.91 days, while that after the implementation of protocol was 7.67 +/- 6.58 days (p < 0.0001). Mortality proportion in patients after implementation of protocol-based ventilator weaning was 0.21 as compared to 0.37 in the control group (p=0.0153). The daily cost of patient care for the LTAC while on mechanical ventilation was $2200/day per patient while it was $ 1400/day per patient while not on mechanical ventilation leading to significant cost savings.

**Conclusion:**

Protocol-driven liberation from mechanical ventilation in LTAC by RT can significantly decrease the duration of a mechanical ventilator, leading to decreased mortality and cost savings.

## 1 INTRODUCTION

More than 21 days of mechanical ventilation for at least six hours a day is referred to as Prolonged Mechanical Ventilation (PMV) [1, 2]. It is estimated that 4 to 13 percent of patients initiated on mechanical ventilation fail to wean and require prolonged mechanical ventilation [2, 3]. PMV not only increases the morbidity and mortality amongst the patients but also incurs a huge economic burden on the healthcare system [3]. PMV also signifies that there is a failure of complete resolution of the pathology which was the original indication for endotracheal intubation. Alternatively, it might also be due to the development of new medical problems while on a mechanical ventilator. Mostly, it is a combination of multiple factors that inhibit early liberation from mechanical ventilation. When patients or their healthcare proxy opt to continue mechanical ventilation, it results in the transfer of the patient to Long-Term Acute Care (LTAC) facility.

## 2 OBJECTIVE

The primary aim of our study was to determine if protocol-driven weaning from mechanical ventilator by Respiratory Therapist (RT) as a part of the Respiratory Disease Certification Program (RDCP) would result in rapid weaning from mechanical ventilation. We also studied the cost-effectiveness of protocol-based weaning from MV by RT in terms of healthcare expenses related to long-term mechanical ventilation and decreased inpatient mortality on MV in the LTAC setting.

## 3 MATERIALS AND METHODS

We conducted this retrospective case-control study on the patients admitted to one of the LTACs in South Texas. All the patients included in the study were admitted to this LTAC from three community-based hospitals situated in the same city. This study was performed as a Quality Improvement (QI) project and was thus exempt from the approval of the local Institutional Review Board (IRB). The hospital committee also approved this QI project. Medical Records (MR) of patients on mechanical ventilation in the LTAC were reviewed before and after the implementation of protocol-based liberation from the ventilator. RT, as a part of RDCP, drove the weaning protocol. The Control group comprised of patients who were on mechanical ventilation 6 months before the initiation of RDCP. Patients seen during the twelve months after the implementation of RDCP formed the study group. A checkbox based weaning protocol driven by RT was implemented in this study (Table **1A** and **B**). The cost-saving, if any, was computed.

## 4 STATISTICAL ANALYSIS

We used mean ± one standard deviation for the continuous data in our study. For categorical data, we used frequencies and percentages. A Standard T-test was utilized to compare results obtained from the control and study groups. Z score calculation for two-population proportion was also used when relevant. A P-value of less than 0.05 was considered statistically significant.

## 5 RESULTS

There were 58 patients on mechanical ventilation prior to the initiation of protocol-based ventilator weaning, who served as the control group. 140 patients on mechanical ventilation after implementation of the protocol-based ventilator weaning formed the study group.

7 patients from the control group who were put on comfort care were excluded from the study. 27 patients who were put on comfort care in the study group were excluded from the study, and also 2 patients in the study group who had missing data were also excluded from the study. The analysis was performed on 51 patients in the control group who were on mechanical ventilation before the initiation of protocol-based ventilator weaning and 111 patients on mechanical ventilation after implementation of the protocol-based ventilator weaning in the study group. There were 56 female patients (50.45%) in the study group and 23 female patients were (45.09%) in the control group. The mean age for the study group was 67.25 +/- 13.3 years, while that for the control group was 69.15 +/-10.11 years. Time to wean from the mechanical ventilation before the implementation of protocol-driven weaning by RT was 16.76 +/- 18.91 while that after the implementation of protocol was 7.67 +/- 6.58 (p < 0.0001). We did not find any statistically significant correlation based on age, gender, and ethnicity (Table **2**). Mortality proportion in patients after implementation of protocol-based ventilator weaning was 0.21 as compared to 0.37 in the control group (p=0.0153) (Table **2**). None of the patients had to be re-intubated and placed back on mechanical ventilation during our study duration. Simplified Acute Physiology Score (SAPS) II on the first day of admission was calculated for both the control and study groups. All the patients included in the study (both control and study groups) had SAPS II score between 29 to 40 points correlating with mortality prediction of 10 to 25%. The daily cost of patient care for the LTAC while on mechanical ventilation was $2200/day per patient while it was $ 1400/day per patient not on mechanical ventilation. We found that the patients were weaned for an average of 9.09 days earlier with the aid of RT driven protocol-based weaning. This resulted in the total cost savings of $807,192 of the 111 patients included in the study group.

## 6 DISCUSSION

There are no definite and optimal weaning protocols for patients who are in LTAC, which can be used with ample confidence in patients requiring PMV. Certain guidelines were issued by a task force formed by the American College of Chest Physicians (ACCP). These guidelines recommend that the weaning be gradual and carefully monitored process [4]. They have set forth certain criteria that need to be fulfilled before proceeding with ventilator weaning. There has to be an accurate determination of the reversal of the original indication for respiratory failure and endotracheal intubation. PaO_2_/FiO_2_ ratio should be more than 150 with FiO_2_ less than 50% and positive end-expiratory pressure (PEEP) less than 8 cm of H_2_O. pH should be more than 7.25 and there should be no evidence of hemodynamic instability. Finally, there should be enough evidence to determine that patients can initiate an inspiratory effort [2, 4]. A randomized controlled trial pertinent to liberation from mechanical ventilation in patients with PMV revealed earlier weaning from the ventilator in patients receiving unassisted breathing via a tracheostomy collar as compared to those on pressure support [5]. The median time to liberation was 15 days (Interquartile range of 8-25 days) in patients on tracheostomy *versus* 19 days [Interquartile range of 12-31 days] in those on MV with an endotracheal tube. Unfortunately, this study could not reveal the mortality benefit of earlier weaning on 6 and 12 months follow-up [2, 5]. Moreover, it was difficult to extrapolate these findings in a different clinical setting as this trial was strictly conducted in a single long-term facility only.

There have been a few other studies that are akin to our study in terms of utilizing protocol based mechanical ventilation weaning strategy. These studies collectively ascertain the need for frequent reassessments as the core measure to yield better results. Scheinhorn *et al.* implemented a respiratory therapist-driven protocol to extubate or wean patients in a post-intensive care unit (ICU) of an LTAC. They used a Therapist-Implemented Patient-Specific (TIPS) weaning protocol after training the staff and collecting and analyzing a sample data obtained from a pilot study [6]. In this study, a total of 252 patients fulfilling PMV with 9,135 cumulative ventilator days were subjected to ventilator weaning as per TIPS protocol. This cohort of the patient population was compared with 238 patients that were taken care of by the same group of pulmonologist and RT over a period of 2 years before implementation of TIPS. The median time for ventilator weaning was 17 days in the TIPS protocol group *versus* 29 days in the control group (p<0.001) [6]. There was no statistically significant difference in mortality of 27.4% in the TIPS protocol group versus 30.7% in the control group (p=0.10) [6]. Vitacca *et al.* conducted a prospective multicenter randomized controlled study in three different long-term weaning facilities utilizing protocol-based weaning for patients on pressure support ventilation or spontaneous breathing trials. The overall 30-day successful weaning rate was 87% in the protocol-driven group in comparison to 70% in the historical control group. Thus, the use of a well-defined weaning protocol per se was the main reason for earlier weaning from mechanical ventilation irrespective of the model used [7]. Chao *et al.* prospectively evaluated the results of a protocol-based ventilator-weaning study and determined that a conservative threshold for rapid shallow breathing index (RSBI) of less than or equal to 80 can be raised to 97 with equally effective weaning results with the aid of RT driven protocol based weaning [8]. The basic design and results of these studies are in concordance with our study. There are not many larger studies solely dedicated to determining the impact of protocol-based weaning in patients on PMV. Thus, our study will likely trigger an enthusiasm in clinicians and researchers to look further into this concept. We all are aware of frequently encountered medical complications of PMV, which are infections, volume overload, bleeding complications in the trachea, development of pneumothorax, renal failure, and laryngeal edema, amongst others [9, 10]. Efforts should be undertaken to overcome these issues related to PMV.

## 7 LIMITATIONS OF OUR STUDY

We conducted a single-center retrospective case-control study rather than a multicenter clinical trial, which would have allowed a larger study cohort, thereby leading to the powerful impact of the study. It is difficult to determine the generalizability of our findings unless similar studies are concomitantly conducted in several institutions. Our study does show a strong causal relationship, but as a general norm in statistics, conclusions drawn from retrospective case-control studies should be verified and tested by prospective cohort studies.

## CONCLUSION

Prolonged mechanical ventilation leads to decreased overall survival, poor functional status, decreased quality of life, increased chances of further medical complications, and higher utilization of healthcare resources. It also places a tremendous burden on the overall healthcare system. Our study suggests that the implementation of a protocol based weaning mechanism driven by RT in an LTAC can achieve quicker liberation from the ventilator. It can also lead to significant cost savings and decreased mortality. Thus, serious consideration should be given to implementing a protocol-based ventilator weaning system by the RT. Our study provides a basis for larger and well-structured multicenter prospective studies to further establish the benefits of protocol-based mechanical ventilation weaning in LTAC.

## Figures and Tables

**Table 1:** Ventilator weaning protocol utilizing checkboxes.

**A. Appearance/Clinical Signs**
a. No diaphoresis, healthy skin color (no cyanosis)
b. Spontaneous cough and gag reflex
c. No accessory muscle use or paradoxical breathing
d. Clear, improved and adequate breathing sounds
e. Significant improvement or reversal of the underlying disease process
f. Patient opening eyes, able to follow simple commands
**B. Weaning Parameters**
a. FiO2 is equal to or less than 50% and PEEP is less than or equal to +5
b. Total respiratory rate in the range of 12-40 breaths per minute
c. Tidal Volume > 5 ml/kg ideal body weight
d. Vital signs stable
e. Fluid balance stable
f. F/Tidal volume ratio <100
**C. Review of recent laboratory values**
a. Arterial blood gas approaching the patient’s baseline
b. Acid-base balance is corrected (optional)
c. Electrolytes are normal
d. Complete blood count near-normal baseline (optional)
e. Albumin > 2 gram/deciliter (optional)
f. Consult physician on any abnormal laboratory results for further orders. Any recommendations require written orders using appropriate Telephone Order Read Back (TORB) form
* If the patient develops any of the following changes in condition or abnormal findings since this protocol was initially initiated by Physician, call the physician for approval before proceeding
**D. Patients meet Criteria Yes No**
Weaning Guidelines Day#: Date:
a. Ensure protocol is ordered and signed by the physician or the physician has given specific weaning orders.
b. Patients should be stable and comfortable in present settings.
c. Perform a weaning assessment on the second day of admission and daily. If the patient is actively weaning, assessments are done every 4 hours during the day
d. Perform and document weaning parameters on Pressure Support Ventilation (PSV) of at least 5 cm of H2O daily, if stable unless otherwise ordered by the physician
e. Obtain arterial blood gas and consult a physician for further orders if abnormal.
f. Choose weaning plan A, B, or C if ready to wean.
* If the above guidelines are all met, proceed to the next plan.

**Table 1A:** Weaning from Assist Control to Pressure Support. Weaning Time < 1 day.

a. Skip Plan A if a patient is already on PSV/Continuous positive airway pressure (CPAP)
b. Change mode from Assist Control to PSV/CPAP
c. Preset PSV by calculation ¾ of current PIP. Do not exceed 20 cm of H2O. Switch modes. Obtain ABG and consult with a physician for further orders if abnormal.
d. The patient’s respiratory rate should be in the range of 12-40 breaths per minute
e. Tidal volume greater than 5 ml/kg
f. Maintain current minute volume
g. Continue to plan B the same day, if appropriate
h. If unable to maintain the AC mode for 3-5 days, go to plan D and evaluate for intermittent sedation.

**Table 1B:** Wean from Pressure Support to T-Collar. Weaning time 2 to 7 days.

A. Phase I-
a. Reduce pressure support (PS) level by 2 to 4 cm H2O each day as tolerated to maintain desired minute ventilation and respiratory rate.
b. PS is never to be less than 5 cm of H2O unless otherwise ordered by the physician
c. Once the patient is on a PS of 10 cm of H2O and can comfortably sustain an adequate spontaneous respiratory pattern for 4 hours, obtain an ABG, and consult with a physician for further orders.
B. Phase II-
a. Place patient on a T-collar 4 hours on, 4 hours off during the day, rest on PSV of 10 cm of H2O at night.
b. The FiO2 may be increased by 5-10% to maintain Oxygen saturations between 92-95%
c. Passy Muir Valve assessment will be completed by day 3 of T-Collar trials.
d. The patient will be treated by physical/ occupational therapy at the optimum time per collaboration with rehabilitation and respiratory therapy
e. Observe closely for fatigue. The patient will appear to struggle at times. The patient must be allowed to work. This will increase the patient’s endurance.
* Obtain ABG on day 1 and every 3^rd^ day while the patient is on phase II. Inform the physician in case of any abnormal occurrence.
C. Phase III-
a. Place patient on T-Collar for 24 hours and check ABG
b. If ABG is acceptable, go to “Decannulation” Section
c. If ABG is not acceptable or the patient did not maintain 24-hour T-collar, move to Plan C
D. Phase IV-
a. Patients admitted with tracheostomy tubes or weaned off the ventilator without the goal of decannulation
b. Notify pulmonologist that the weaning phase is completed
c. Obtain a physician order to downsize tracheostomy tube to cuffless non-fenestrated

**Table 2:** Table showing basic demographic characteristics and main outcomes of the study.

Variables	Study Group	Control Group	-
Sample size (n)	111	51	-
Sex	Female: 56 (50.5%)	Female: 23 (45.1%)	p =0.26
	Male: 55 (49.5%)	Male: 28 (54.9%)	P=0.26
Mean age (years)	67.25 +/- 13.3	69.15 +/- 10.11	P= 0.67
Ethnicity	Caucasian Americans: 51 (45.9%)	Caucasian Americans: 23 (45.1%)	P=0.46
	Hispanic Americans: 51 (45.9%)	Hispanic Americans: 25 (49%)	P=0.35
	African Americans: 9 (8.2%)	African Americans: 3 (5.9%)	P=0.30
Time to wean ventilator (days)	7.67 +/- 6.58	16.67 +/- 18.91	P<0.001
Mortality proportion	24/111 (0.21)	19/51 (0.37)	P <0.05 (0.0153)
